# *Sirt3* deficiency promotes endothelial dysfunction and aggravates renal injury

**DOI:** 10.1371/journal.pone.0291909

**Published:** 2023-10-10

**Authors:** Anna Pezzotta, Luca Perico, Daniela Corna, Marina Morigi, Giuseppe Remuzzi, Ariela Benigni, Barbara Imberti

**Affiliations:** Istituto di Ricerche Farmacologiche Mario Negri IRCCS, Bergamo, Italy; Boston University School of Medicine, UNITED STATES

## Abstract

Sirtuin 3 (SIRT3), the main deacetylase of mitochondria, modulates the acetylation levels of substrates governing metabolism and oxidative stress. In the kidney, we showed that SIRT3 affects the proper functioning of high energy-demanding cells, such as tubular cells and podocytes. Less is known about the role of SIRT3 in regulating endothelial cell function and its impact on the progression of kidney disease. Here, we found that whole body *Sirt3*-deficient mice exhibited reduced renal capillary density, reflecting endothelial dysfunction, and VEGFA expression compared to wild-type mice. This was paralleled by activation of hypoxia signaling, upregulation of HIF-1α and Angiopietin-2, and oxidative stress increase. These alterations did not result in kidney disease. However, when *Sirt3*-deficient mice were exposed to the nephrotoxic stimulus Adriamycin (ADR) they developed aggravated endothelial rarefaction, altered VEGFA signaling, and higher oxidative stress compared to wild-type mice receiving ADR. As a result, ADR-treated *Sirt3*-deficient mice experienced a more severe injury with exacerbated albuminuria, podocyte loss and fibrotic lesions. These data suggest that SIRT3 is a crucial regulator of renal vascular homeostasis and its dysregulation is a predisposing factor for kidney disease. By extension, our findings indicate SIRT3 as a pharmacologic target in progressive renal disease whose treatments are still imperfect.

## Introduction

Sirtuin 3 (SIRT3), the major mitochondrial deacetylase, regulates several important biological functions that encompass cell metabolism, mitochondrial dynamics, and antioxidant activity [1]. It has been recognized that SIRT3 expression is crucial for the maintenance of the appropriate physiological status of organs with elevated metabolic rates through supporting cellular stress resistance. The kidney is one of the main energy-demanding organs in the human body [2], primarily because of its role in incessantly filtering blood, regulating the balance of electrolytes and acid–base homeostasis, reabsorbing nutrients, and blood pressure control.

As a result, SIRT3 dysregulation has been reconducted to the propensity toward kidney dysfunction and the development of more severe diseases [1]. Indeed, mice with global deficiency of *Sirt3* developed a more severe disease in an experimental mouse model of acute kidney injury (AKI) induced by cisplatin [3]. Also, studies evidenced that global *Sirt3* deficiency aggravates contrast‑induced AKI [4] and hypertension-induced renal fibrosis [5]. On the other hand, SIRT3 has been demonstrated being protective in AKI, as pharmacological manipulations that increased SIRT3 in wildtype mice protected animals through the preservation of antioxidant activity and mitochondrial integrity [3, 6].

On the top of that, SIRT3 protein expression has been shown to decrease during lifetime [7] and loss of *Sirt3* is associated with premature ageing [8, 9]. The kidney, along with the heart and brain, is one of the main organs susceptible to age-related diseases, translating into increased vulnerability to chronic kidney diseases (CKD) in the elderly population [10].

Consistently, it has been suggested that SIRT3 activation contributes to decrease renal inflammation and fibrosis during chronic renal damage [11]. As well, renal *Sirt3* expression and activity was found reduced in a model of type 2 diabetic nephropathy accompanied by the increase in oxidative stress and mitochondrial damage [12]. Moreover, whole body *Sirt3*-deficiency has been shown to accelerate kidney disease in response to nutrient overloads [13].

In this latter study, one of the most striking results was that *Sirt3*-deficient mice exhibited endothelial alterations, in terms of vascular rarefaction [13]. Consistently, studies in cardiac tissue showed indeed that endothelial-specific *Sirt3* ablation caused microvascular dysfunction and reduced capillary density [14]. Cardiac vascular rarefaction in the absence of *Sirt3* was associated with impaired cardiac recovery after myocardial ischemia [14, 15].

While these studies clearly suggest the critical role of SIRT3 on endothelial cell function, scarce literature is available documenting the underlying molecular mechanism and the actual impact of SIRT3 deficiency on endothelial dysfunction and on the progression of diseases affecting the kidney. Srivastava *et al*. showed that SIRT3 protein is expressed by kidney endothelial cells and its expression is significantly reduced in endothelial cells isolated from diabetic kidneys [16]. Endothelial cell dysfunction has been recognized as being one of the earliest pathogenic alterations in the glomeruli, even preceding that of podocytes, during CKD [17]. The specific mechanism by which endothelial dysfunction occurs during kidney disease is not completely understood and dissecting the molecular determinants in the alteration of endothelial cell function during early stages of the pathology could be relevant to identify novel therapeutic strategies to limit disease initiation and progression.

The aim of the present study is to evaluate the impact of global *Sirt3* deficiency on renal endothelial cell phenotype and to investigate whether SIRT3 deficiency impacts on endothelial dysfunction and on the severity of renal injury induced by the nephrotoxic agent Adriamycin (ADR).

## Materials and methods

### Animal experiments

All procedures involving animals were performed in accordance with institutional guidelines in compliance with national (D.L.n.26, March 4, 2014), and international laws and policies (directive 2010/63/EU on the protection of animals used for scientific purposes). This study was approved by the Institutional Animal Care and Use Committees of Istituto di Ricerche Farmacologiche Mario Negri IRCCS and by the Italian Ministry of Health (approval number 16/2017-PR). This study was carried out in compliance with the ARRIVE guidelines [18]. Every effort was made to minimize stress, discomfort, and pain of the experimental animals.

The animals were randomly allocated to experimental groups. No inclusion or exclusion parameters were used in our studies. Investigators were not blinded to treatments, but no subjective assessments were made.

Whole-body *Sirt3*^-/-^ deficient mice, generated in a mixed genetic background, were provided by Professor Frederick Alt, Harvard Medical School, Boston, MA, USA [19]. As control, their C57BL6x129 wild-type (WT) littermates were used. All mice were maintained in a specific pathogen–free facility with a 12-hour light/dark cycle with free access to standard diet and water.

Chronic kidney disease was induced in two-month-old male animals through a single dose of Adriamycin (ADR, 18 mg/kg; Pfizer Italia S.r.l) by tail-vein injection with small size needles (30G) to reduce the suffering of the animals. No analgesia was needed since no mice exhibited signs of distress. The following groups of mice were studied: group 1, WT+saline (n = 4); group 2, WT+ADR (n = 4); group 3, *Sirt3*^-/-^+saline (n = 4); *Sirt3*^-/-^+ADR (n = 6).

For urinary protein/creatinine ratio, proteinuria was determined weekly with the Coomassie method using Cobas Mira auto-analyzer (Roche Diagnostic Systems). Briefly, in a buffered solution, proteins in the urine sample reacted with Coomasie Blue and the intensity of color was quantified by the auto analyzer. Urinary creatinine concentration was measured using the enzymatic method with Cobas Mira auto-analyzer. Renal function was assessed as blood urea nitrogen (BUN) using the Reflotron test (Roche Diagnostics) following manufacturer’s instructions. All animals were anesthetized with isoflurane (induction 4±5%, maintenance 2±3%, Piramal Critical Care Italia SpA) prior to blood withdrawal, and all efforts were made to minimize suffering. At 7 weeks after disease induction, mice were euthanised through CO_2_ inhalation and their kidneys collected and processed for analysis.

### Renal histology

Duboscq-Brazil-fixed, 3 μm paraffin-embedded kidney sections were stained with periodic acid-Schiff reagent. At least 50 glomeruli were examined for each animal. The number of glomeruli exhibiting glomerulosclerosis was expressed as percentage. Tubular damage (dilation and atrophy) was quantified in at least 15 fields (magnification, X100) and was graded between 0 and 4 (0, no changes; 1, changes affecting 25% of the sample; 2, changes affecting >25–50% of the sample; 3, changes affecting >50–75% of the sample; 4, changes affecting >75–100% of the sample). Image analyses were performed in a blinded fashion.

### Immunostaining studies on kidney sections

For the immunofluorescence analysis of kidney sections, 3-μm periodate-lysine paraformaldehyde (PLP)-fixed cryosections were air dried, washed with PBS 1× and incubated with 1% bovine serum albumin (BSA) to block non-specific sites. The following primary antibodies were used: rat anti-MECA-32 (Developmental studies hybridoma bank, University of Iowa, 1:50), rabbit anti-VEGFA (Abcam, ab52917, 1:100), mouse anti-nestin (BD Biosciences, BD556309, 1:100), rabbit anti-WT1 (Santa Cruz Biotechnology, sc-192, 1:50), rabbit anti-fibronectin (Abcam, ab2040, 1:600), rabbit anti-CD31 (Abcam, ab2836, 1:50) followed by the appropriate FITC or Cy3-conjugated secondary antibodies (Jackson ImmunoResearch Laboratories). Nuclei were stained with 4’,6-diamidino-2-phenylindole (DAPI, Sigma Aldrich) and the renal structure with fluorescein wheat germ agglutinin (WGA, Vector Laboratories, FL-1021). Negative controls were obtained by omitting primary antibodies on adjacent sections.

To detect WT1, antigen retrieval was performed in citrate buffer 10 mmol/L (pH 6.0) at boiling temperature for 20 minutes, followed by 20-minute incubation with citrate buffer at room temperature to enhance the reactivity of antibodies to antigens. Finally, slides were mounted using Dako Fluorescence Mounting Medium (DAKO) and examined with an inverted confocal laser microscope (Leica TCS SP8).

Estimation of glomerular volume (V_G_) was performed using a computer-based image analysis system on digitized histological sections of fluorescein-WGA-labeled glomeruli. Exact enlargement in micrometers per pixel of digital images was calculated from images of a reference grid digitized at the same resolution. The outline of the minimal polygon around the glomerular tuft area was manually traced, and its surface area automatically measured, in the same glomeruli examined for counting WT1-positive cells in each tissue section. Mean value of V_G_ was then calculated as previously reported [20].

The average number of podocytes per glomerulus was determined in 30 glomeruli for each animal through the stereological method of particle density on digital images acquired with confocal inverted laser microscope [20]. Briefly, the volume density of the podocytes (N_V_) in glomerular tuft volume was estimated with the formula N_V_ = N_A_/D where N_A,_ podocyte nuclear profile area density, was calculated as the ratio between the numbers of podocyte nuclear profiles and the glomerular profile area in each glomerulus. D is the average diameter of podocyte nuclei that we estimated from major and minor axis of cell nuclear sections, calculated as previously reported [20].

To quantify fibronectin deposition, immunofluorescence images were acquired at confocal microscope. Specifically, to quantify glomerular fibronectin, n = 15 glomeruli *per* section were acquired. Glomerular profiles were traced manually based on WGA staining of renal tissues and glomerular area was calculated. For quantification of tubular fibronectin, n = 15 randomly selected HPF were acquired. Digitized images were dichotomized using a threshold for staining (ImageJ software), and the values were expressed as the percentage of staining per glomerulus or per total area of the acquired field, as appropriate.

The same procedure was applied on dichotomized images to quantify the expression of markers (MECA-32, VEGFA, CD31) analysed by immunofluorescence. Image analyses were performed in a blinded fashion.

### Immunoperoxidase analysis

Formalin-fixed, 3-μm paraffin embedded kidney sections were incubated with Peroxidazed 1 (Biocare Medical, PX968) to quench endogenous peroxidase, after antigen retrieval in a decloaking chamber with Rodent decloaker buffer. After blocking for 30 minutes with Rodent Block M (Biocare Medical, RBM961G), sections were incubated with rabbit anti-VEGFR2 (Cell Signalling, 2479, 1:100), rabbit anti-HIF-1α (Santa Cruz Biotechnology, sc-10790, 1:25), rabbit anti-Nitrotyrosine antibody (Merck, 06–284, 1:100), and rabbit anti-VEGFR1 (Abcam, ab2350, 1:200) followed by Rabbit on Rodent HRP-Polymer (Biocare Medical, RMR622G) for 30 minutes at room temperature. Staining was visualized using betazoid 3,3′diaminobenzidine chromogen kit solutions (Biocare Medical, BDB2004H). Slides were counterstained with Mayer’s hematoxylin (Bio Optica), mounted with Eukitt mounting medium and finally observed using light microscopy (ApoTome, Axio Imager Z2, Zeiss). Negative controls were obtained by omitting the primary antibody on adjacent sections. Glomerular VEGFR1 and VEGFR2 stainings were quantified by threshold and the positive glomerular areas were expressed as a percentage of the total area (ImageJ Software). At least 15–20 glomeruli/section for each animal were analysed randomly. Tubular VEGFR2, HIF-1α, and nitrotyrosine stainings were quantified with a semiquantitative score between 0 and 3 (0: absent staining, 1: weak staining, 2: moderate staining, 3: intense staining). At least 10–15 fields/section for each animal were randomly analysed (original magnification, x 400). Image analyses were performed in a blinded fashion.

### Protein extraction and Western blot analysis

For total protein extraction, excised renal tissues were washed twice in normal 0.9% (w/v) sodium chloride solution and lysed by homogenization in CelLytic MT (Sigma-Aldrich, C3228) supplemented with protease inhibitor cocktail (Sigma-Aldrich, P8340) and phosphatase inhibitors (Sigma-Aldrich, 4906845001). After homogenization, the sample lysates were centrifuged 16,000×*g* for 10 min at 4 °C and protein concentration of total protein extracts was determined using DC assay (Bio-Rad Laboratories, 5000111). Equal amounts of proteins (30 μg) were separated on 12% sodium dodecyl sulfate (SDS)-polyacrylamide gel electrophoresis under reducing conditions and transferred to nitrocellulose membranes (Bio-Rad Laboratories, 1704159). After blocking with 5% BSA (Sigma-Aldrich) in tris-buffered saline (TBS) supplemented with 0.1% Tween-20 (Sigma-Aldrich), membranes were incubated with the following antibodies: rabbit anti-VEGFA (Cell Signalling, 50661, 1:1000), mouse anti-Angpt-2 (R&D system, MAB098, 1:1000), rabbit anti-SOD2^AcK68^ antibody (Abcam, ab137037, 1:1000), and goat anti-SIRT3 (Abcam, ab118334, 1:1000). On the same membranes, mouse anti-α-tubulin (Sigma-Aldrich, T9026, 1:1000) was used as sample loading control. The signals were visualized on Odyssey FC Imaging System (LiCor, Lincoln, Nebraska, USA) by infrared (IR) fluorescence using a goat anti-rabbit IRDye 680LT (LiCor, 926–68023, 1:1000) or a goat anti-mouse IRDye 800CW (LiCor, 926–32210; 1:1000), as appropriate. Enhanced chemiluminescence-Western Blotting Detection Reagent (Pierce, ThermoFisher, A38554) using donkey anti-goat horseradish peroxidase (HRP)-conjugated secondary antibodies (Sigma-Aldrich, AP180P, 1:20000) secondary antibody. Bands were quantified by densitometry using the Image Studio Lite 5.0 (LiCor) software. All uncropped gels are provided in S1 Raw images.

### Statistical analysis

Results were expressed as mean ± standard error of the mean (SEM). Data analysis was performed using Graph Pad Prism software (Graph Pad). The sample size for each analysis is indicated in the corresponding Figure legend. Comparisons were made using Student’s t-test or one-way ANOVA corrected with Bonferroni or Tukey’s multiple comparisons post hoc test, as appropriate. The statistical significance was defined as P<0.05.

## Results

### *Sirt3*-deficient mice exhibit glomerular and peritubular capillary rarefaction

**Fig 1:**
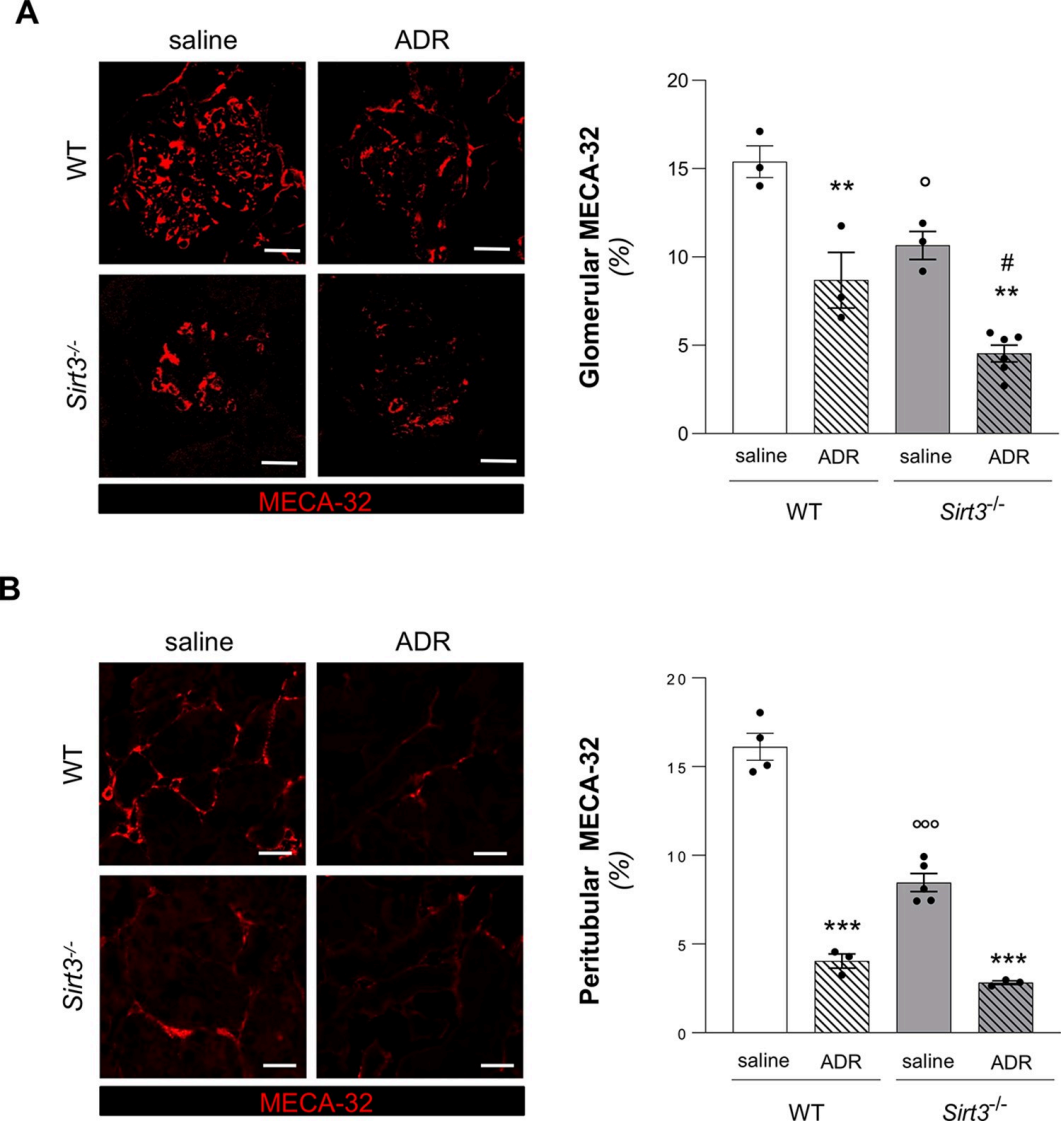
*Sirt3* deficiency induces endothelial loss in the kidney and worsens ADR-induced glomerular vascular rarefaction. (**A**) Representative images and quantification of glomerular endothelial cells expressed as the percentage of glomerular area positive for MECA-32 staining in WT and *Sirt3*^*-/-*^ mice, treated with saline or ADR (n = 3 mice for all groups except for n = 6 mice in *Sirt3*^*-/-*^+ADR). (**B**) Representative images and quantification of peritubular microvascular endothelial cells expressed as the percentage of MECA-32 positive area/high-power field in WT and *Sirt3*^*-/-*^ mice, treated with saline or ADR, evaluated at 7 weeks (n = 4 mice, WT + saline; n = 3 mice, WT+ADR and *Sirt3*^*-/-*^+ADR; n = 5 mice, *Sirt3*^*-/-*^+saline). Data represent mean ± SEM and were analyzed by one-way ANOVA followed by Tukey’s multiple comparisons test. **P<0.01, and ***P<0.001 *vs* corresponding saline; °P<0.05, and °°°P<0.001 *vs* WT+saline; ^#^P<0.05 *vs* WT+ADR. Scale bars, 20 μm.

To understand whether *Sirt3* deficiency could impact on pathological changes in renal microvascular compartment, we stained renal tissues at 7 weeks with the murine endothelial marker MECA-32 and quantified glomerular endothelial density. As shown in **Fig 1A,** glomerular capillary density was significantly reduced in *Sirt3*^-/-^ mice as compared to WT mice. The lack of SIRT3 severely impacted on peritubular capillary loss (**Fig 1B**). These results have been confirmed by using the endothelial cell marker, CD31. As shown in **S1 Fig**, *Sirt3*^-/-^ mice exhibited capillary rarefaction in both glomerular and peritubular areas as compared to WT thus indicating that *Sirt3* deficiency induce intrinsic vascular changes of the kidney.

### *Sirt3*-deficient mice exhibit altered VEGFA and VEGFR2 expression in the glomeruli

**Fig 2:**
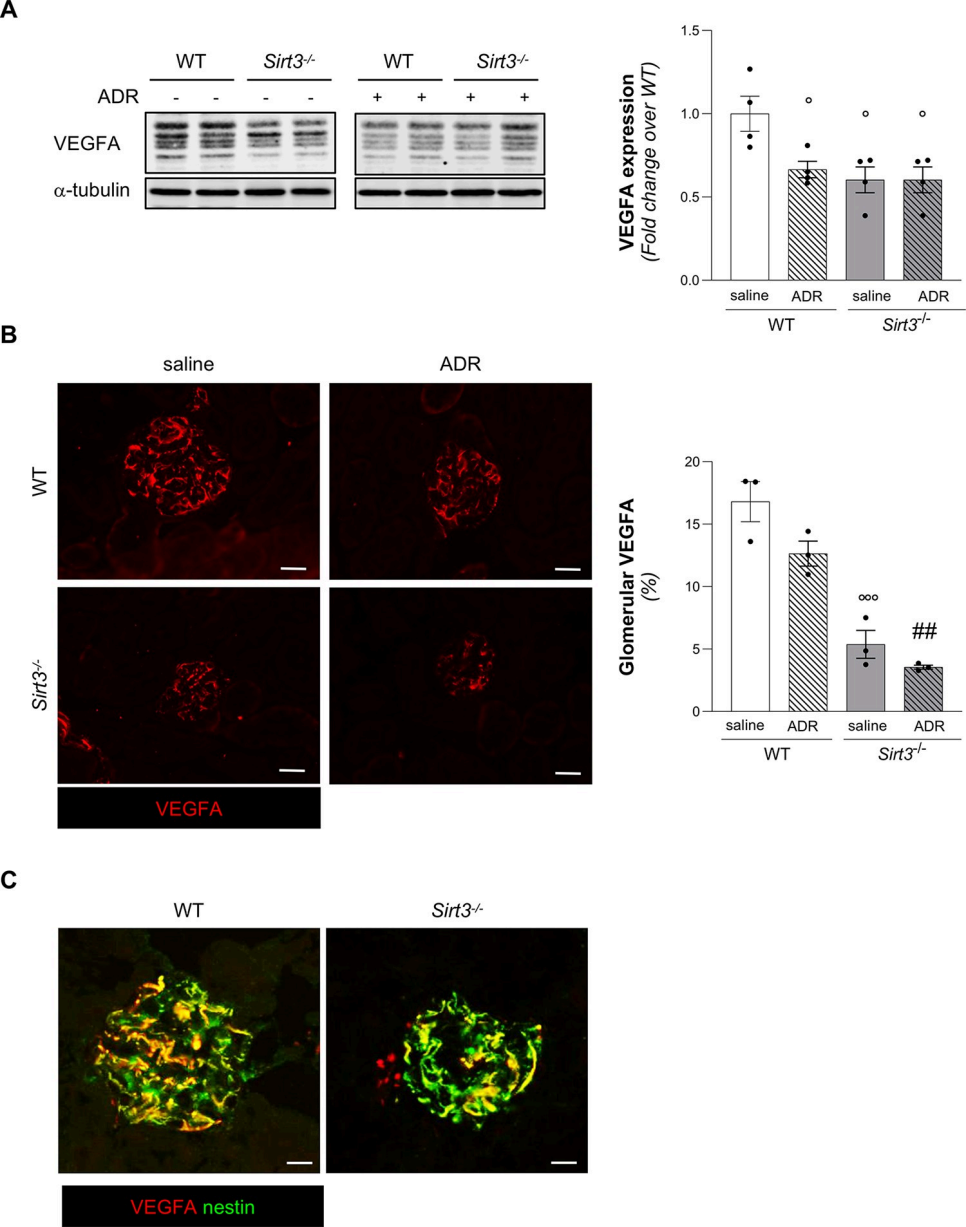
*Sirt3* deficiency severely impairs VEGFA expression and further reduces glomerular VEGFA upon ADR treatment. (**A**) Representative Western Blot and quantification of VEGFA in WT and *Sirt3*^*-/-*^ mice, treated with saline or ADR (n = 4 mice per groups). (**B**) Representative images and quantification of glomerular VEGFA in WT and *Sirt3*^*-/-*^ mice after 7 weeks receiving saline or ADR (n = 3 mice for group). Scale bars, 20 μm. (**C**) Representative images of double staining for VEGFA (red) and nestin (green) in WT and *Sirt3*^*-/-*^ mice. Scale bars, 10 μm. Data represent mean ± SEM and were analyzed by one-way ANOVA followed by Tukey’s multiple comparisons test. °P<0.05, and °°°P<0.001 *vs* WT+saline; ^###^P<0.001 *vs* WT+ADR.

To investigate the potential cause of capillary reduction observed in *Sirt3*^-/-^ mice, we studied the proangiogenic molecule vascular endothelial growth factor A (VEGFA). VEGFA plays a relevant role in regulating glomerular structure and function, both in normal and pathogenic conditions [21]. The protein expression of VEGFA was then quantified on total renal extracts by Western Blot analysis, revealing that *Sirt3*^-/-^ mice exhibited significant reduction of VEGFA (**Fig 2A**). Given the relevance of VEGFA expression in maintaining glomerular cell wellness, we quantified VEGFA expression per glomerulus and found that glomerular VEGFA was reduced in *Sirt3*^*-/-*^ as compared to WT mice (**Fig 2B**). By double staining with VEGFA and the podocyte marker nestin, we observed that in both WT and *Sirt3*^-/-^ mice, VEGFA is mainly localized in podocytes (**Fig 2C**).

**Fig 3:**
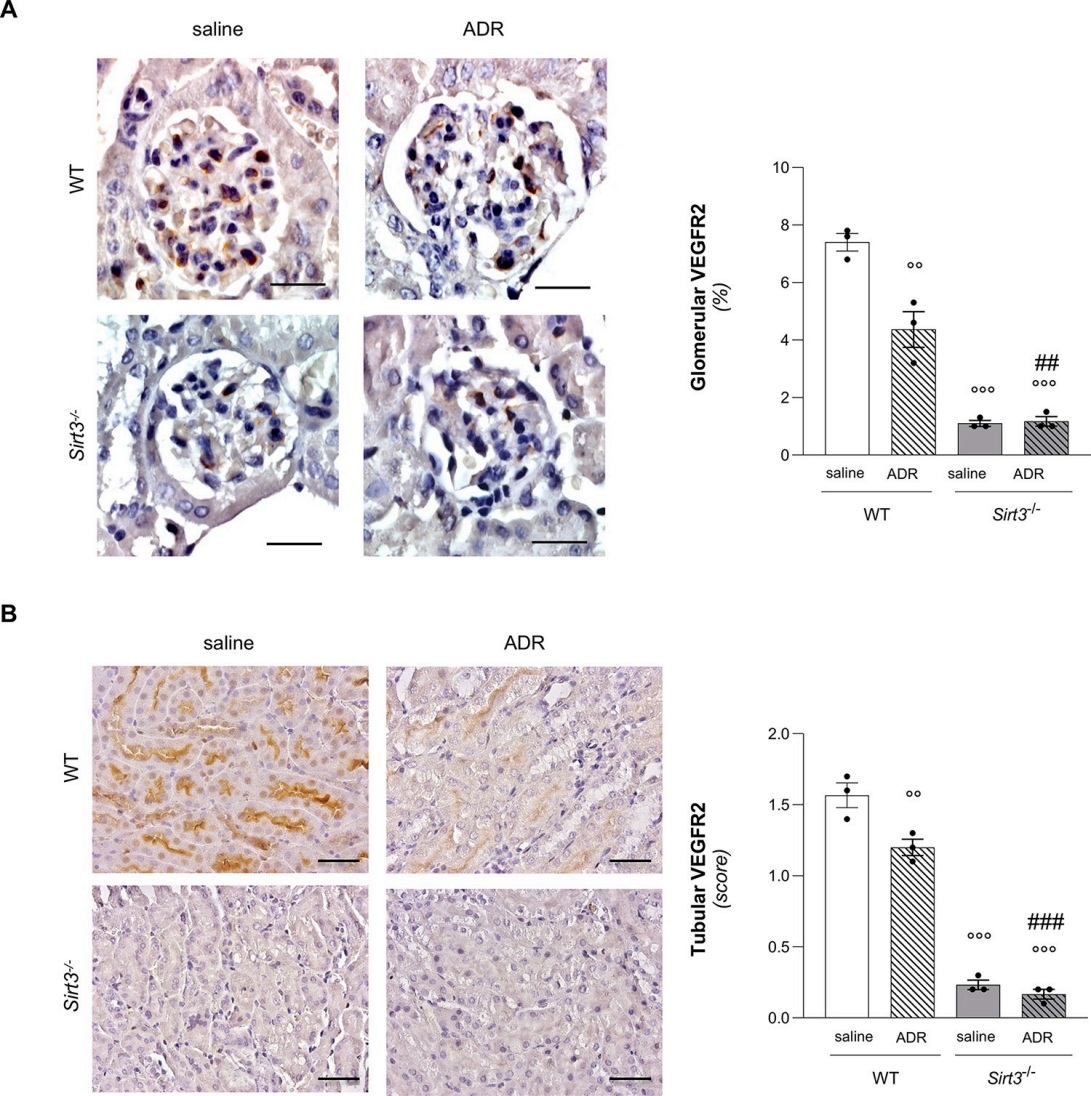
*Sirt3* deficiency severely impairs VEGFR2 expression, which is further reduced upon ADR treatment. Representative images and quantification of (**A**) glomerular and (**B**) tubular VEGFR2 in WT and *Sirt3*^*-/-*^ mice, treated with saline or ADR, evaluated at 7 weeks (n = 3 mice for group). Data represent mean ± SEM and were analyzed by one-way ANOVA followed by Tukey’s multiple comparisons test. °°P<0.01, and °°°P<0.001 *vs* WT+saline; ^##^P<0.01, and ^###^P<0.001 *vs* WT+ADR. Scale bars, 20 μm in panel A and 50 μm in panel B.

The proangiogenic action of VEGFA is mediated by its two cognate receptors, VEGFR1 and VEGFR2. We therefore investigated whether glomerular expression of VEGF receptors could be affected by *Sirt3* deficiency. By immunohistochemical analysis, we found that VEGFR1 was not affected by *Sirt3* deficiency, as revealed by comparable expression levels of VEGFR1 in WT and *Sirt3*^-/-^ mice (**S2 Fig**). Conversely, when we analyzed VEGFR2 in mice receiving saline, we found that the *Sirt3*^-/-^ mice had significantly reduced expression of VEGFR2 as compared to WT mice in both at glomerular (**Fig 3A**) and tubular (**Fig 3B**) level by immunohistochemical analysis.

### Renal vascular rarefaction in *Sirt3*-deficient mice is associated with hypoxic and pro-oxidative states

**Fig 4:**
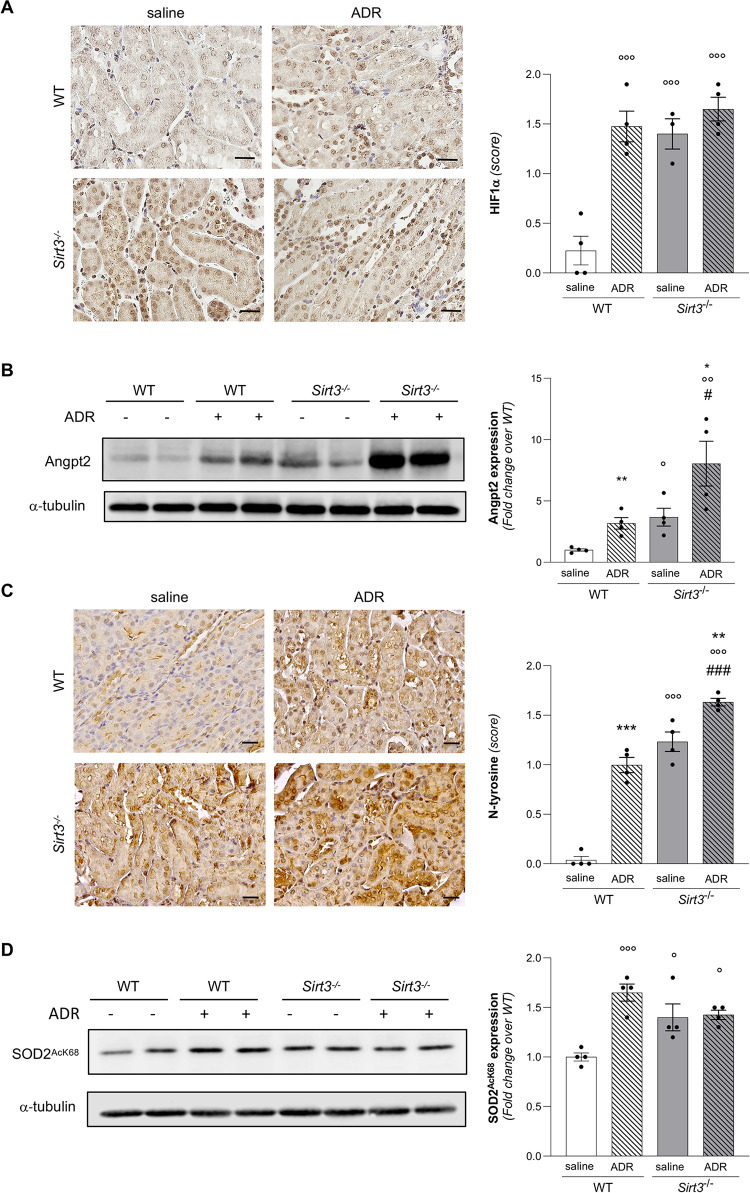
*Sirt3* deficiency increases the hypoxic and oxidative state of the kidney and exacerbates ADR-induced oxidative stress. (**A**) Representative images and quantification of HIF-1α expression in WT and *Sirt3*^*-/-*^ mice after 7 weeks receiving saline or ADR (n = 4 mice for all groups except for n = 3 mice in *Sirt3*^*-/-*^+saline). (**B**) Representative Western Blot and quantification of Angpt-2 in WT and *Sirt3*^*-/-*^ mice, treated with saline or ADR, (n = 4 mice for groups). (**C**) Representative images and quantification of nitrotyrosine (N-tyrosine) staining in WT and *Sirt3*^*-/-*^ mice after 7 weeks receiving saline or ADR (n = 4 mice for all groups). (**D**) Representative Western Blot and quantification of acetylated SOD2 at lysine 68 (SOD2 AcK) in WT and *Sirt3*^*-/-*^ mice, treated with saline or ADR (n = 4 mice for all groups). Data represent mean ± SEM and were analyzed by one-way ANOVA followed by Tukey’s multiple comparisons test. °P<0.05, °°P<0.01, and °°°P<0.001 *vs* WT+saline. *P<0.05, **P<0.01, and ***P<0.001 *vs* corresponding saline; ^#^P<0.05, and ^###^P<0.001 *vs* WT+ADR. Scale bars, 20 μm.

The reduction of oxygen supply, as a consequence of reduced capillary density, may affect the expression of hypoxia-inducible factor 1 (HIF-1α), which is involved in the transcriptional response to hypoxia [22]. By examining the expression of HIF-1α with immunohistochemistry, we found that *Sirt3*^*-/-*^ mice exhibited a significant increase in the expression of HIF-1α compared to WT mice **(Fig 4A**), suggesting that the lack of *Sirt3* affects renal tissue oxygenation.

Among the multiple target genes of HIF-1α [23], angiopoietin-2 (Angpt-2) is a well-recognized vascular destabilizing factor that also has a role in the progression of CKD [24]. Based on these findings, we then investigated whether *Sirt3* deficiency could affect Angpt-2 expression in the kidney. As shown by Western Blot analysis, Angpt2 expression increased significantly in *Sirt3*-deficient mice as compared to WT control mice (**Fig 4B**).

Hypoxic conditions are also associated with higher intracellular levels of oxidative stress, particularly in the form of increased reactive oxygen species (ROS), produced mainly at the mitochondrial level [25, 26]. In addition, SIRT3 holds a pivotal role in the regulation of mitochondrial ROS production by regulating the activity of antioxidant enzymes [27]. Given these premises, we sought to investigate if a disbalance in production or elimination of ROS is a hallmark feature associated with endothelial dysfunction in *Sirt3*^*-/-*^ mice. To this aim, we performed an immunohistochemical analysis by staining renal tissues for nitrotyrosine (NT), a marker of oxidative stress, which identifies protein nitration. The results showed that in both glomerular and tubular compartments *Sirt3*^*-/-*^ mice exhibited higher oxidized products compared to WT (**Fig 4C**).

In order to substantiate the finding of oxidative stress, we performed Western blot analysis of the anti-oxidant enzyme superoxide dismutase 2 (SOD2). In particular, we investigated the expression levels of SOD2 acetylated at lysine 68 (SOD2^Kac68^), which inversely correlates with SOD2 activity and is a specific target of SIRT3 deacetylase activity [28]. In this context, we found significant higher SOD2^Kac68^ expression in *Sirt3*^*-/-*^ kidneys (**Fig 4D**), suggesting decreased anti-oxidant activity.

### *Sirt3*-deficient mice do not spontaneously develop kidney disease

In order to assess whether *Sirt3* deficiency was enough to cause renal disease, we examined renal function parameters in *Sirt3*^-/-^ mice at 2 and 12 months of age. As shown in **Table 1**, *Sirt3*^-/-^ mice exhibited urinary protein excretion and serum BUN levels comparable to those measured in matched WT mice. Moreover, systemic blood pressure was comparable in both groups over time (2 months, WT 102.9±2.4 and *Sirt3*^-/-^ 102.8±3.4 mmHg; 12 months, WT 111.8±2.7 and *Sirt3*^-/-^ 106.3±3.4 mmHg; mean ± SEM). Histological analysis of renal tissues of *Sirt3*^-/-^ mice did not reveal signs of glomerular or tubulo-interstitial damage (**Table 1)**. Representative histological images are shown in **S3 Fig**.

**Table 1:** Renal function parameters and histology.

		WT		*Sirt3* ^*-/-*^	
		2 months	12 months	2 months	12 months
Proteinuria (*mg/day*)		1.12±0.10	1.13±0.13	0.82±0.12	0.84±0.05
BUN (*mg/dl*)		21.3±2.2	20.7±1.1	22.2±2.4	22.5±2.4
Glomerulosclerosis (*%*)		0	0	0	0
Tubular damage (*score*)		0	0	0	0.1±0.1

Data represent mean ± SEM; Wild type (WT).

These data indicate that *Sirt3* deficiency is not a condition that leads in adulthood to the development of overt renal disease.

### *Sirt3*-deficient mice exhibit worsened vascular rarefaction, VEGFA loss and oxidative stress upon ADR-induced renal injury

In order to study whether *Sirt3* deficiency holds a detrimental role in the development of CKD, we induced nephropathy by Adriamycin (ADR) injection in *Sirt3*^-/-^ mice [29]. Eight-week-old mice were injected with ADR at a dose that induces mild nephropathy in WT mice to allow to better detect the differences caused by *Sirt3* deficiency. In WT mice, renal injury induced by ADR is associated with a reduction of SIRT3 protein expression, as shown by Western Blot analysis (**S4 Fig**).

In the glomerular compartment, treatment with ADR in WT mice induced a significant reduction in endothelial density (**Fig 1A and S1A Fig**). Of note, the endothelial rarefaction observed in *Sirt3*^-/-^ kidneys further worsened when *Sirt3*^-/-^ mice were treated with ADR (**Fig 1A and S1A Fig**). In the tubular compartment, a decrease in glomerular endothelium was induced by ADR in WT mice (**Fig 1B and S1B Fig)** as well in *Sirt3*^*-/-*^ mice, administration of ADR significantly reduced peritubular capillary density compared to *Sirt3*^-/-^ mice receiving saline (**Fig 1B and S1B Fig**).

Then, we analysed the expression of VEGFA protein by Western Blot in total renal extracts. These results highlighted that ADR induced a significant decrease of VEGFA in WT mice. *Sirt3*^-/-^ mice receiving ADR exhibited levels of VEGFA similar to *Sirt3*^-/-^ mice receiving saline, both lower as compared to WT mice receiving saline (**Fig 2A**). We also characterized the expression of VEGFA protein in glomeruli by quantification of immunofluorescence staining and we found that ADR induced a non-significant reduction in VEGFA, which was instead remarkably reduced in *Sirt3*^-/-^ mice receiving ADR to a comparable level of that observed in *Sirt3*-deficient mice given saline (**Fig 2B**). VEGFR1 expression did not differ in WT and *Sirt3*^-/-^ mice with progressive ADR nephropathy (**S2 Fig**). Otherwise, in WT mice receiving ADR the expression of VEGFR2 significantly dropped to levels comparable of those observed in *Sirt3*^-/-^ mice receiving saline (**Fig 3A and 3B**). Administration of ADR to *Sirt3*-deficient mice did not further reduce the already low levels of VEGFR2 found in *Sirt3*^-/-^ mice receiving saline (**Fig 3A and 3B**).

By examining the expression of HIF-1α, we found that ADR significantly induced the expression HIF-1α in WT mice to similar levels of *Sirt3*^-/-^ mice receiving saline (**Fig 4A**). Administration of ADR to *Sirt3*-deficient mice was not associated with boosted expression of HIF-1α compared to *Sirt3*^-/-^ mice receiving saline **(Fig 4A**). Consistently, by Western Blot analysis we observed that ADR significantly increased the expression of Angpt-2 compared to WT mice receiving saline (**Fig 4B**). Moreover, *Sirt3*-deficient mice given ADR exhibited further heightening Angpt-2 expression compared to *Sirt3*-mice receiving saline (**Fig 4B**). Collectively, these data suggest that the hypoxic signaling triggered by the lack of *Sirt3* is so pronounced that it cannot be further aggravated by the superimposition of ADR-induced kidney damage.

Furthermore, WT mice receiving ADR showed the rise of the nitrotyrosine staining to a similar extent of *Sirt3*-deficient mice (**Fig 4C**). Finding that nitrotyrosine staining was even higher in *Sirt3*^*-/-*^ mice receiving ADR compared to KO mice receiving saline (**Fig 4C**) indicates that mice lacking *Sirt3* experience excessive ROS production in the kidney of mice with induced nephropathy. Consistently, SOD2^Kac68^ was significantly increased upon ADR challenge in both WT and *Sirt3*^*-/-*^ mice (**Fig 4D**).

### *Sirt3*-deficient mice exhibit exacerbated proteinuria and podocyte loss upon ADR-induced renal injury

**Fig 5:**
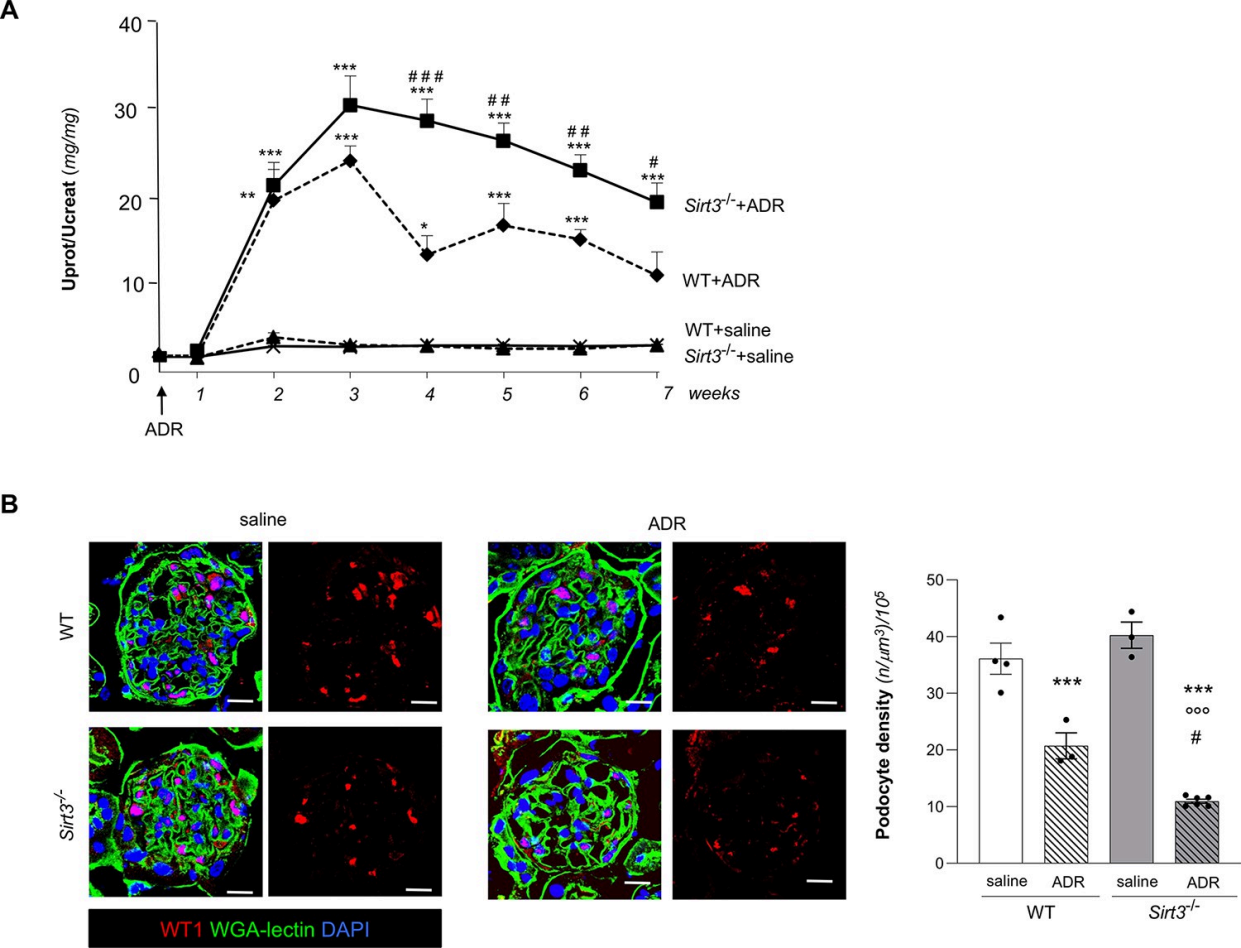
*Sirt3* deficiency aggravates ADR-induced urinary protein excretion and podocyte loss. (**A**) Urinary protein/creatinine ratio (Uprot/Ucreat) measured during time in WT and *Sirt3*^*-/-*^ mice receiving saline or ADR (n = 4 mice for all groups except for n = 6 mice in *Sirt3*^*-/-*^+ADR). Data represent mean ± SEM and were analyzed with ANOVA corrected with Bonferroni coefficient. *P<0.05, **P<0.01, and ***P<0.001 *vs* corresponding saline; ^#^P<0.05, ^##^P<0.01, and ^###^P<0.001 *vs* WT+ADR at the corresponding time. (**B**) Representative images showing WT1-positive podocytes (red), WGA lectin (green) and DAPI (blue) staining in WT and *Sirt3*^*-/-*^ mice after 7 weeks receiving saline or ADR, and quantification of podocyte density (n = 4 mice, WT + saline; n = 3 mice, WT+ADR and *Sirt3*^*-/-*^ mice + saline; n = 6 mice *Sirt3*^*-/-*^ mice + ADR). Data represent mean ± SEM and were analyzed by one-way ANOVA followed by Tukey’s multiple comparisons test. ***P<0.001 *vs* corresponding saline; °°°P<0.001 vs WT+saline; ^#^P<0.05 *vs* WT+ADR. Scale bars, 20 μm.

We then investigated whether endothelial changes observed in renal tissues of *Sirt3*^-/-^ mice could be associated with alterations in protein glomerular filtration. Compared to animals receiving vehicle, in WT and *Sirt3*^-/-^ mice, ADR induced a significant increase in proteinuria after 2 weeks, which peaked at 3 weeks and declined over time (**Fig 5A**). ADR injection yielded a higher urinary protein/creatinine ratio in *Sirt3*^-/-^ mice compared to WT animals, and there was a statistically significant difference starting at 4 weeks (**Fig 5A**).

In order to characterize the damage induced by ADR treatment, we analyzed kidney tissues at sacrifice, at 7 weeks after ADR injection. Since podocyte injury is a critical step towards glomerular lesions in ADR-induced nephropathy and podocyte damage is involved in the loss of the permselective function of the glomerular filtration barrier, WT and *Sirt3*^-/-^ renal tissues were stained with the podocyte marker WT1 and glomerular podocyte density was measured. Podocyte number/glomerulus and glomerular volume have been quantified and results are shown in **S5 Fig**. Of note, *Sirt3*-deficient mice exhibited reduced glomerular volume as compared to WT mice (**S5 Fig**). Podocyte density was comparable in *Sirt3*^-/-^ and WT mice receiving saline (**Fig 5B**). Following exposure to ADR, WT mice underwent a remarkable podocyte loss (30% decrease) and, notably, the decrease in podocyte density was exacerbated in *Sirt3*^-/-^ mice (72% decrease) (**Fig 5B**).

These findings indicate that *Sirt3*-deficient mice experience severe podocyte loss with consequent protein leakage in case of nephrotoxic insult.

### *Sirt3*-deficient mice exhibit increased glomerular fibrosis in ADR-induced renal injury

**Fig 6:**
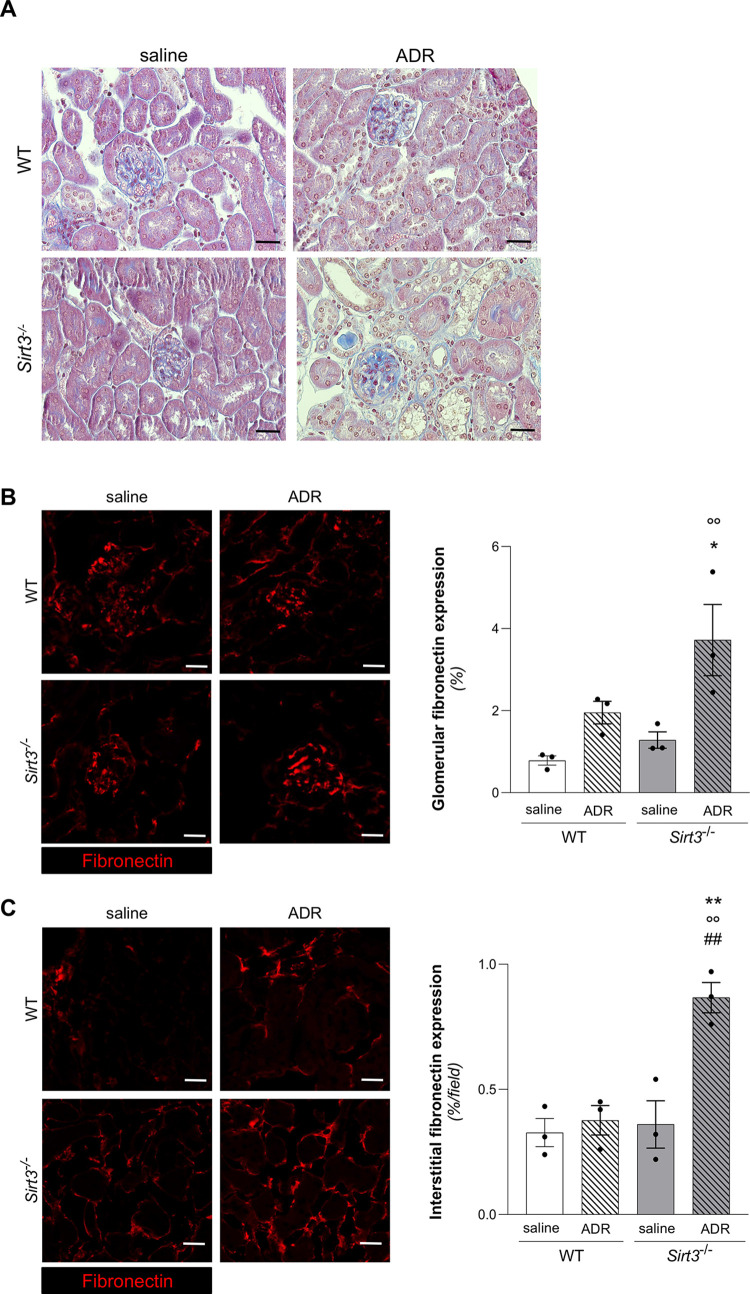
*Sirt3* deficiency increases ADR-induced fibrosis. (**A**) Representative images of Masson’s trichrome stain of kidney tissue of WT and *Sirt3*^*-/-*^ mice, treated with saline or ADR, evaluated at 7 weeks. (**B, C**) Representative images and quantification of fibronectin staining in glomerular and interstitial area in renal section in WT and *Sirt3*^*-/-*^ mice after 7 weeks receiving saline or ADR (n = 3 mice for all groups). Data represent mean ± SEM and were analyzed by one-way ANOVA followed by Tukey’s multiple comparisons test. *P<0.05, and **P<0.01 *vs* corresponding saline; °°P<0.01 *vs* WT+saline; ^##^P< 0.01 *vs* WT+ADR. Scale bars, 20 μm.

The extent of renal damage was characterized by the analysis of extracellular matrix deposition. The analysis of Masson’s trichrome staining showed glomerular fibrotic lesions only in *Sirt3*^-/-^ mice exposed to ADR (**Fig 6A**). This result was corroborated by the significant increase at glomerular level in fibronectin deposition in *Sirt3*^-/-^ mice receiving ADR (**Fig 6B**), which exhibited as well an increase in fibronectin deposition also within tubular areas (**Fig 6C**). These data indicate that *Sirt3* deficient mice exhibit higher susceptibility to develop kidney fibrotic lesions upon nephrotoxic hit.

## Discussion

In the present study, we provided compelling evidence that SIRT3 holds a critical role in regulating the proper homeostasis of the renal vasculature and that the altered phenotype of endothelial cells in whole body *Sirt3*-deficient mice concurs to the progression and the severity of renal injury induced by Adriamycin administration.

The first, unanticipated finding of this study is that SIRT3 plays a major role in renal endothelial cell function, as evidenced by impairment of glomerular and tubular endothelium in *Sirt3*-deficient mice. The striking reduction in renal capillaries in *Sirt3*-deficient mice could be the result of impaired angiogenesis, which is mediated by the interplay between VEGFA and its cognate receptor VEGFR2. It has been shown that the loss of *Sirt3* limits angiogenesis of endothelial progenitor cells *via* reduced VEGFA and VEGFR2 expression [30]. *Sirt3*-deficient mice showed blunted recovery from myocardial infarction and brain ischemic stroke due to their impaired VEGF-dependent angiogenic capacity [30, 31]. Consistently, our data show that VEGFA and VEGFR2 are significantly reduced in the glomeruli of *Sirt3*-deficient mice, even prior to kidney damage induction. Mechanistically, the ability of *Sirt3* to regulate VEGFA/VEGFR axis could be ascribed to its activity on AKT and extracellular signal‐regulated kinases (ERK) signaling pathways [31]. Indeed, the lack of *Sirt3* led to a decrease in AKT and ERK phosphorylation, which were accompanied by reduced expression of VEGFA [31]. Collectively, these data highlight an unprecedented role of SIRT3 in preserving the glomerular endothelium homeostasis by regulating the cellular crosstalk between podocytes and endothelial cells. For this reason, modulating SIRT3 by means of specific activators, such as honokiol [32], may represent a promising intervention to preserve glomerular endothelial cell function during kidney disease [12, 33].

The most likely functional consequence of the severe vascular rarefaction that we observed in our model is the impairment of oxygen supply, with activation of hypoxia signaling. HIF-1α, the prototypical factor involved in the transcriptional responses to cellular hypoxia, was upregulated in both the glomerular and tubular compartments at nuclear levels in *Sirt3*-deficient mice prior to eliciting kidney damage, and was not further increased upon the induction of renal injury. Our results are in line with the findings that microvascular loss is the primary event that induces HIF-1α expression and VEGFA downregulation during ADR nephropathy [34].

In this context, the activation of hypoxic signaling in renal tissues of *Sirt3*-deficient mice was accompanied by a parallel increase, even prior to ADR-induced damage, in the expression of Angiopoietin-2 (Angpt-2), a multimeric protein involved in vascular remodeling, which was found to be induced in mice with CKD in injured tubular cells [35] and also in renal tubular and endothelial cells of mice with AKI [36]. The biologic effects of Angpt-2, which include vessel leakage, inflammation, and destabilization [37], are dependent on levels of VEGFA, such that vessel regression occurs when VEGFA is lacking [38]. That *Sirt3* deficiency could be responsible for Angpt-2 induction was demonstrated in a model of sepsis showing that knockout of *Sirt3* upregulated Angpt-2, accompanied by a severe reduction in pericyte/endothelial cell coverage and exacerbation of vascular leakage [39]. Moreover, upregulation of Angpt-2 expression in *Sirt3* deficient mice, accelerated angiotensin-II-induced endothelial dysfunction in the kidney and exacerbated ROS formation [5].

Oxidative stress actually plays a role in our experimental setting as demonstrated by the high levels of protein nitration in *Sirt3*-deficient mice that may contribute to tissue dysfunction even in ADR unchallenged mice. Oxidative stress is one of the mechanisms responsible for endothelial dysfunction [40]. Several studies have highlighted the role of *Sirt3* in protecting from oxidative damage by its deacetylating activity of mitochondrial enzymes resulting in the regulation of ROS production and clearance [27]. Dikalova and colleagues showed that SIRT3 dictates vascular dysfunction by regulating oxidative stress *via* SOD2 [41], a direct target of SIRT3 deacetylase activity in the adult kidney [42, 43]. In our setting, increased acetylation levels of SOD2 were found in *Sirt3*-deficient mice, thus implying decreased anti-oxidant capacity of the kidney and further increased oxidative stress. Studies also reported that *Sirt3* deficiency induces endothelial dysfunction in different model of disease [13, 44, 45].

Despite all these major changes in endothelial physiology, *Sirt3*-deficient mice did not manifest overt renal dysfunction, thus suggesting that the kidney can counteract the hypoxic and pro-oxidant milieu induced by *Sirt3* deficiency. In line with this notion, recent studies have demonstrated the presence of hypoxia in renal tissue preceding the presence of markers of kidney injury [46, 47]. Our findings substantiate this hypothesis and indicate that, at least in our experimental setting, the hypoxic and pro-oxidant conditions observed in the absence of SIRT3 are not sufficient to induce overt renal disease, unless these mice are exposed to a second hit.

To test this possibility, we superimposed renal injury induced by treatment with the nephrotoxic drug ADR on *Sirt3* deficiency. In this occurrence, we found that lack of *Sirt3* predisposed mice to exacerbated albuminuria and increased histological lesions in the kidney, characterized by extracellular matrix accumulation. These alterations were accompanied by exacerbated microvascular loss and oxidative stress in the kidney. Indeed, ADR induced a further impairment of vascular density and provoked a remarkable injury and loss of glomerular podocytes. This finding may imply that the concomitant alteration of endothelial cells and podocytes is required to induce full-blown alteration in the glomerular filtration barrier, thus affecting the progression of glomerular injury during kidney disease [48, 49]. In our experimental setting, finding that *Sirt3* deficiency does not affect podocyte density may be the underlying cause for which *Sirt3*^*-/-*^ mice do not show overt renal function impairment. However, the impaired vascular density being present before induction of renal injury in *Sirt3*-deficient mice could predispose mice to increased severity of ADR-induced damage, as vascular dysfunction is a central event in development of kidney disease [50–53]. These findings are also consistent with our previous study indicating that the alterations occurring during kidney development in *Sirt3*-deficient mice may lead to increased susceptibility to renal disease later in life [42].

Collectively, all these data indicate that SIRT3 has a role in regulating endothelial cell wellness through the modulation of VEGFA/HIF-1α signaling. This altered endothelial phenotype could contribute to the increased susceptibility to renal damage in *Sirt3*-deficient mice. These findings are relevant considering that SIRT3 decreases during lifetime [7] and loss of *Sirt3* is associated with premature ageing [8, 9]. Given that the prevalence of chronic nephropathy rises with age [54], age-related reduction of SIRT3 could be responsible for endothelial cell dysfunction, reduction of the vascular network and more severe renal damage. Therefore, upregulating SIRT3 may represent a novel approach for supporting endothelial cell function and preventing major complications of kidney disease.

## Supporting information

S1 Fig *Sirt3* deficiency induces endothelial loss in the kidney and worsens ADR-induced glomerular vascular rarefaction. (**A**) Representative immunofluorescence images and quantification of glomerular endothelial cells expressed as the percentage of glomerular area positive for CD31 staining in WT and *Sirt3*^*-/-*^ mice, treated with saline or ADR (n = 3 mice for all groups). (**B**) Representative immunofluorescence images and quantification of peritubular microvascular endothelial cells expressed as the percentage of CD31 positive area/high-power field in WT and *Sirt3*^*-/-*^ mice, treated with saline or ADR (n = 3 mice for all groups). Data represent mean ± SEM and were analyzed by one-way ANOVA followed by Tukey’s multiple comparisons test. *P<0.05, and **P<0.01 *vs* corresponding saline; °°P<0.01, and °°°P<0.001 *vs* WT+saline; ^#^P<0.05, and ^##^P<0.01 *vs* WT+ADR. Scale bars, 20 μm.
(TIF)

S2 Fig *Sirt3* deficiency and/or ADR treatment do not affect VEGFR1 expression. Representative immunohistochemical images of VEGFR1 and quantification in WT and *Sirt3*^*-/-*^ mice receiving saline or ADR (n = 4 mice, WT+saline and *Sirt3*^*-/-*^+ADR; n = 3 mice, WT+ADR and *Sirt3*^*-/-*^+saline). Data represent mean ± SEM and were analyzed by one-way ANOVA followed by Tukey’s multiple comparisons test. Scale bar, 20 μm.
(TIF)

S3 Fig *Sirt3*-deficient mice do not spontaneously develop renal damage. Histological analysis of kidneys from WT and Sirt3^-/-^ mice at 2 and 12 months of age stained with periodic acid–Schiff. Scale bars, 50 μm.
(TIF)

S4 Fig ADR treatment significantly downregulates renal SIRT3 expression. Representative Western Blot and quantification of SIRT3 protein expression in WT mice treated with saline or ADR (n = 4 mice per group). Data represent mean ± SEM and were analysed with Student’s t-test. °°°P<0.001 vs WT + saline.
(TIF)

S5 Fig *Sirt3* deficiency affects podocyte number and glomerular volume. (**A**) Quantification of the number of podocytes per glomerulus in WT and *Sirt3-*deficient mice receiving saline or ADR (n = 4 mice, WT + saline; n = 3 mice, WT+ADR and *Sirt3*^*-/-*^ mice + saline; n = 6 mice *Sirt3*^*-/-*^ mice + ADR). (**B**) Quantification of the glomerular volume in WT and *Sirt3* deficient mice receiving saline or ADR (n = 4 mice, WT + saline; n = 3 mice, WT+ADR and *Sirt3*^*-/-*^ mice + saline; n = 6 mice *Sirt3*^*-/-*^ mice + ADR). Data represent mean ± SEM and were analyzed by one-way ANOVA followed by Tukey’s multiple comparisons test. **P<0.01 *vs* corresponding saline; °P<0.05, and °°°P<0.001 *vs* WT+saline.
(TIF)

S1 Raw images Uncropped blot images included in the manuscript’s main figures and supplemental figures. All images were acquired on Odyssey FC Imaging System (LiCor, Lincoln, Nebraska, USA). In each blot, α-tubulin was used as sample loading control. Molecular weights (MW) are reported for each gel and expressed in kilo Dalton (kDa). X indicates lanes not included in the final figures. Abbreviations: WT, wild type mice; *Sirt3*^*-/-*^, *Sirt3* knockout mice; ADR, adriamycin; VEGFA, vascular endothelial growth factor A; Angpt2, angiopoietin 2; SOD2^AcK68^, SOD2 acetylated at lysine 68; L-SIRT3, long SIRT3 isoform; S-SIRT3, short SIRT3 isoform (mitochondria).
(PDF)
