# Supplementary figures and images for: Sirt3 deficiency promotes endothelial dysfunction and aggravates renal injury
Source: PLoS One. 2023 Oct 10;18(10):e0291909. doi: 10.1371/journal.pone.0291909 (PMC10564163; doi:10.1371/journal.pone.0291909)

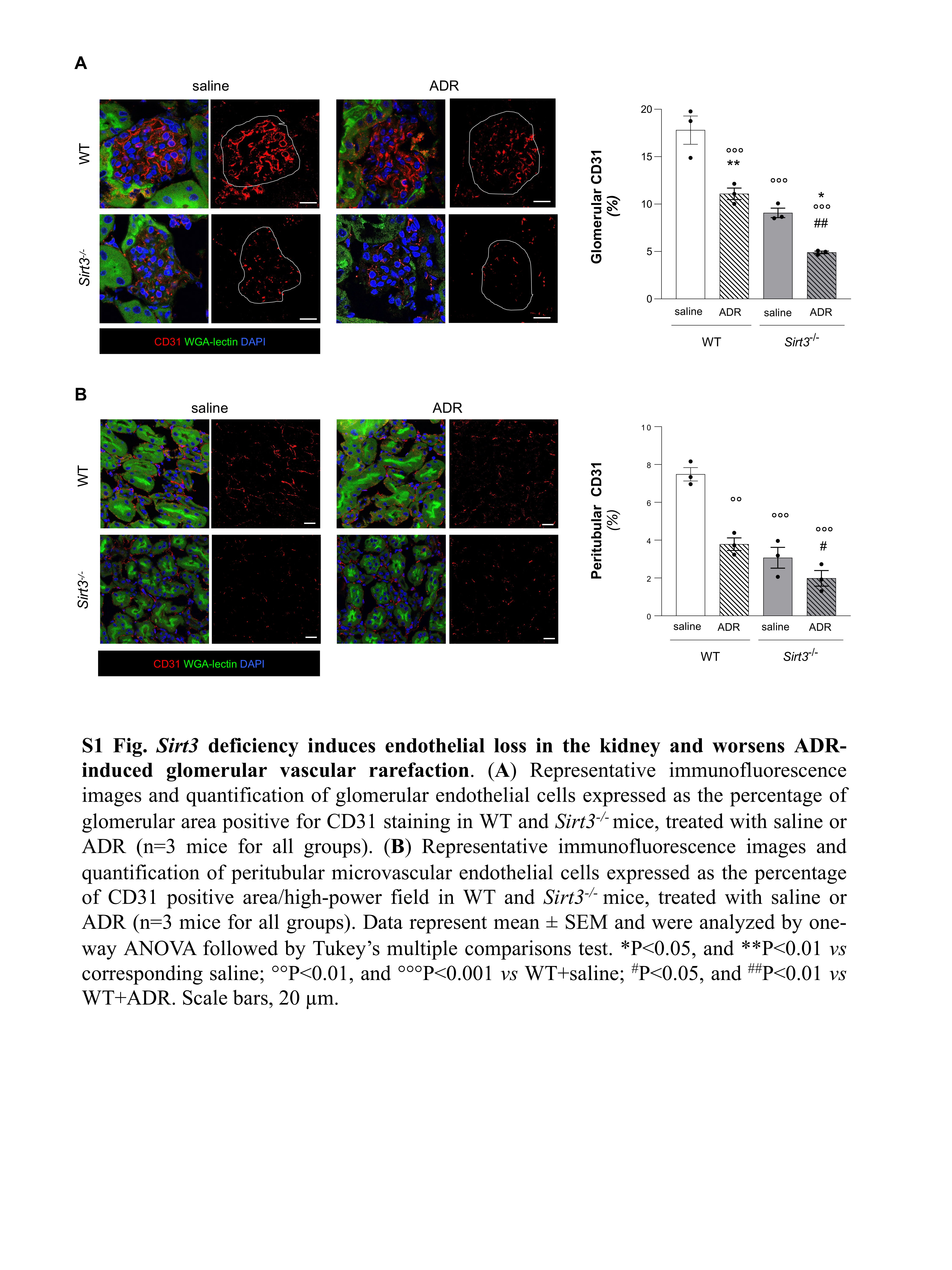

Supplement: S1 Fig — (A) Representative immunofluorescence images and quantification of glomerular endothelial cells expressed as the percentage of glomerular area positive for CD31 staining in WT and Sirt3-/- mice, treated with saline or ADR (n = 3 mice for all groups). (B) Representative immunofluorescence images and quantification of peritubular microvascular endothelial cells expressed as the percentage of CD31 positive area/high-power field in WT and Sirt3-/- mice, treated with saline or ADR (n = 3 mice for all groups). Data represent mean ± SEM and were analyzed by one-way ANOVA followed by Tukey’s multiple comparisons test. *P<0.05, and **P<0.01 vs corresponding saline; °°P<0.01, and °°°P<0.001 vs WT+saline; #P<0.05, and ##P<0.01 vs WT+ADR. Scale bars, 20 μm. (TIF) [file pone.0291909.s001.tif]

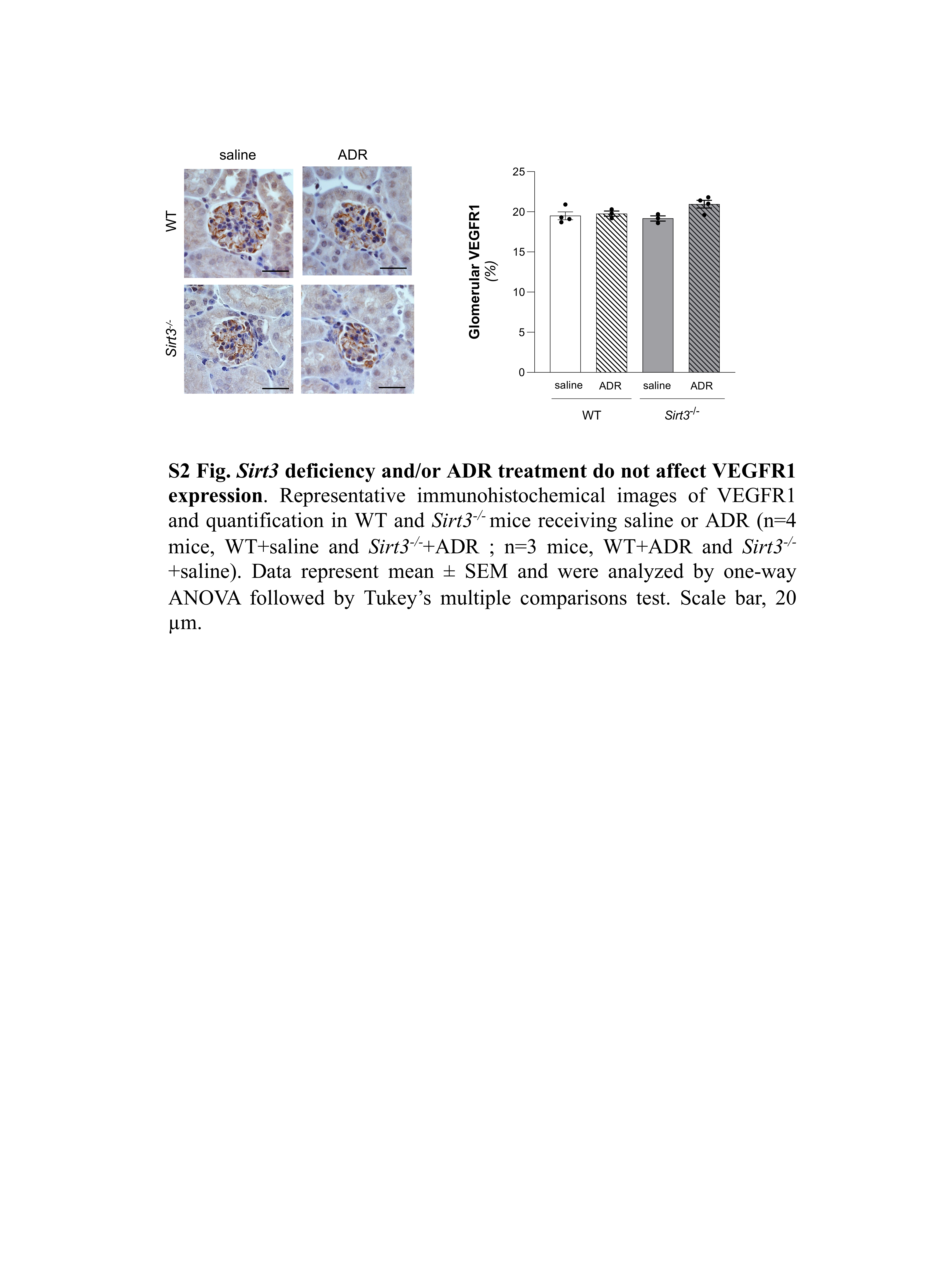

Supplement: S2 Fig — Representative immunohistochemical images of VEGFR1 and quantification in WT and Sirt3-/- mice receiving saline or ADR (n = 4 mice, WT+saline and Sirt3-/-+ADR; n = 3 mice, WT+ADR and Sirt3-/-+saline). Data represent mean ± SEM and were analyzed by one-way ANOVA followed by Tukey’s multiple comparisons test. Scale bar, 20 μm. (TIF) [file pone.0291909.s002.tif]

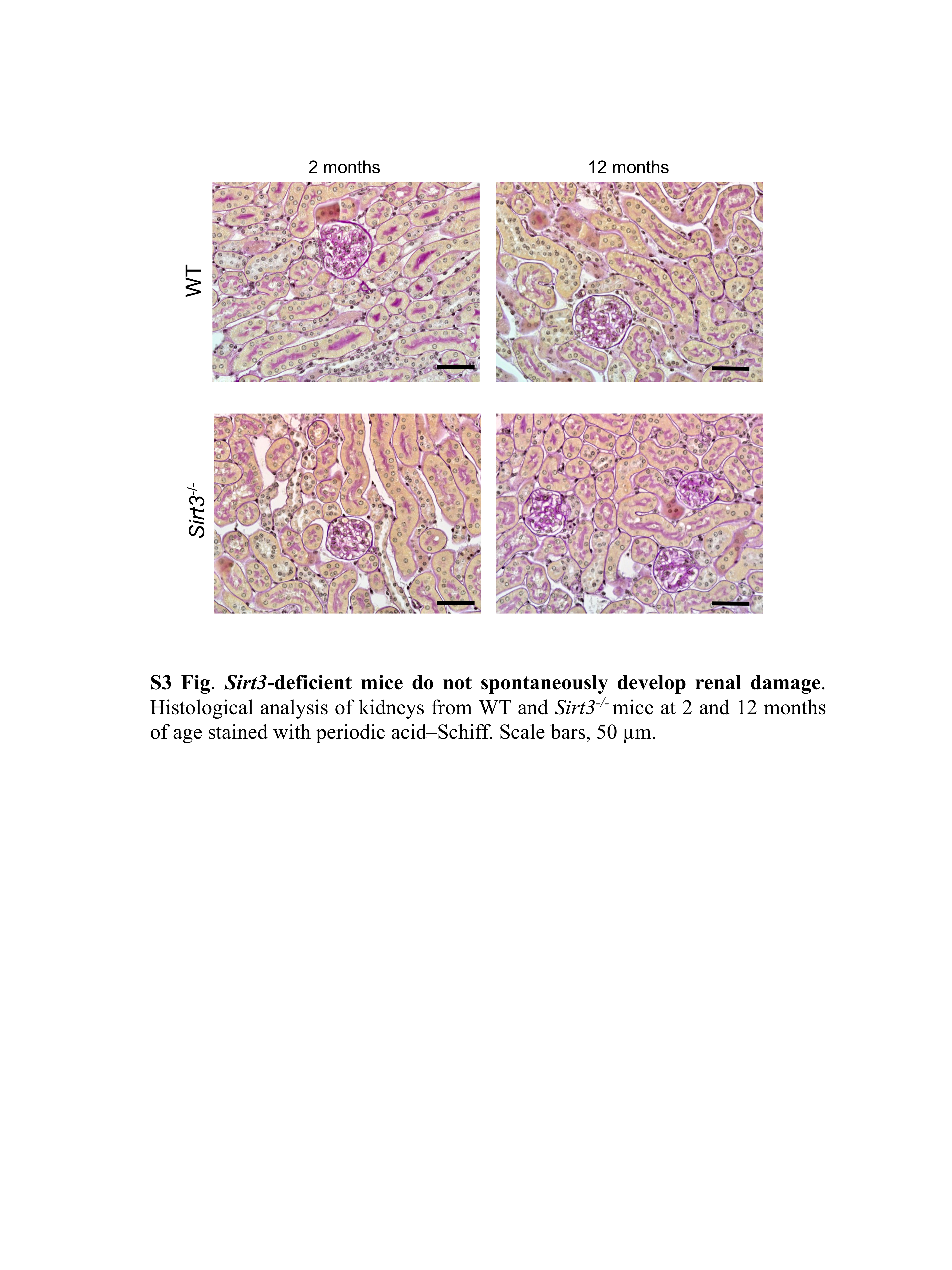

Supplement: S3 Fig — Histological analysis of kidneys from WT and Sirt3-/- mice at 2 and 12 months of age stained with periodic acid–Schiff. Scale bars, 50 μm. (TIF) [file pone.0291909.s003.tif]

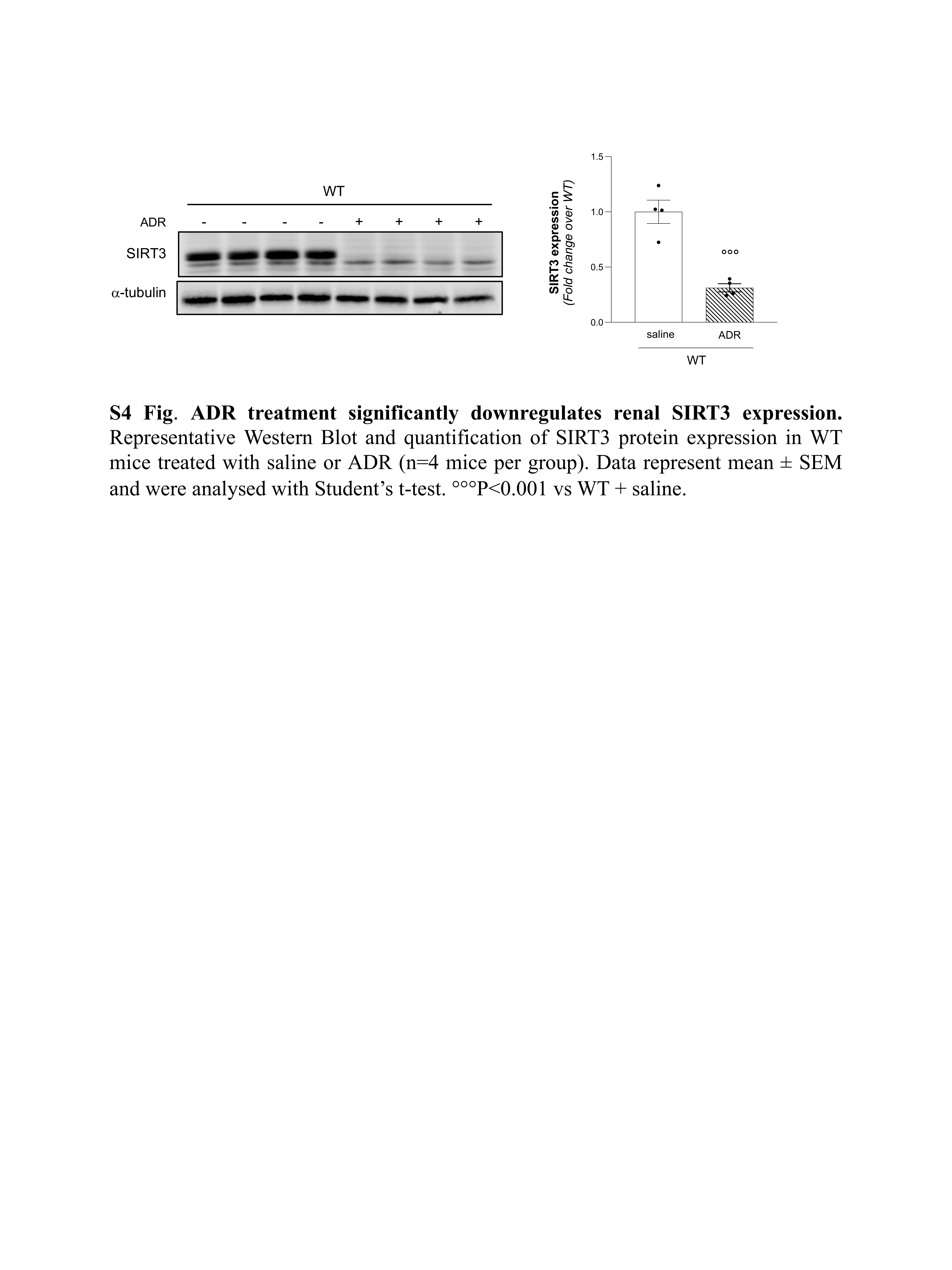

Supplement: S4 Fig — Representative Western Blot and quantification of SIRT3 protein expression in WT mice treated with saline or ADR (n = 4 mice per group). Data represent mean ± SEM and were analysed with Student’s t-test. °°°P<0.001 vs WT + saline. (TIF) [file pone.0291909.s004.tif]

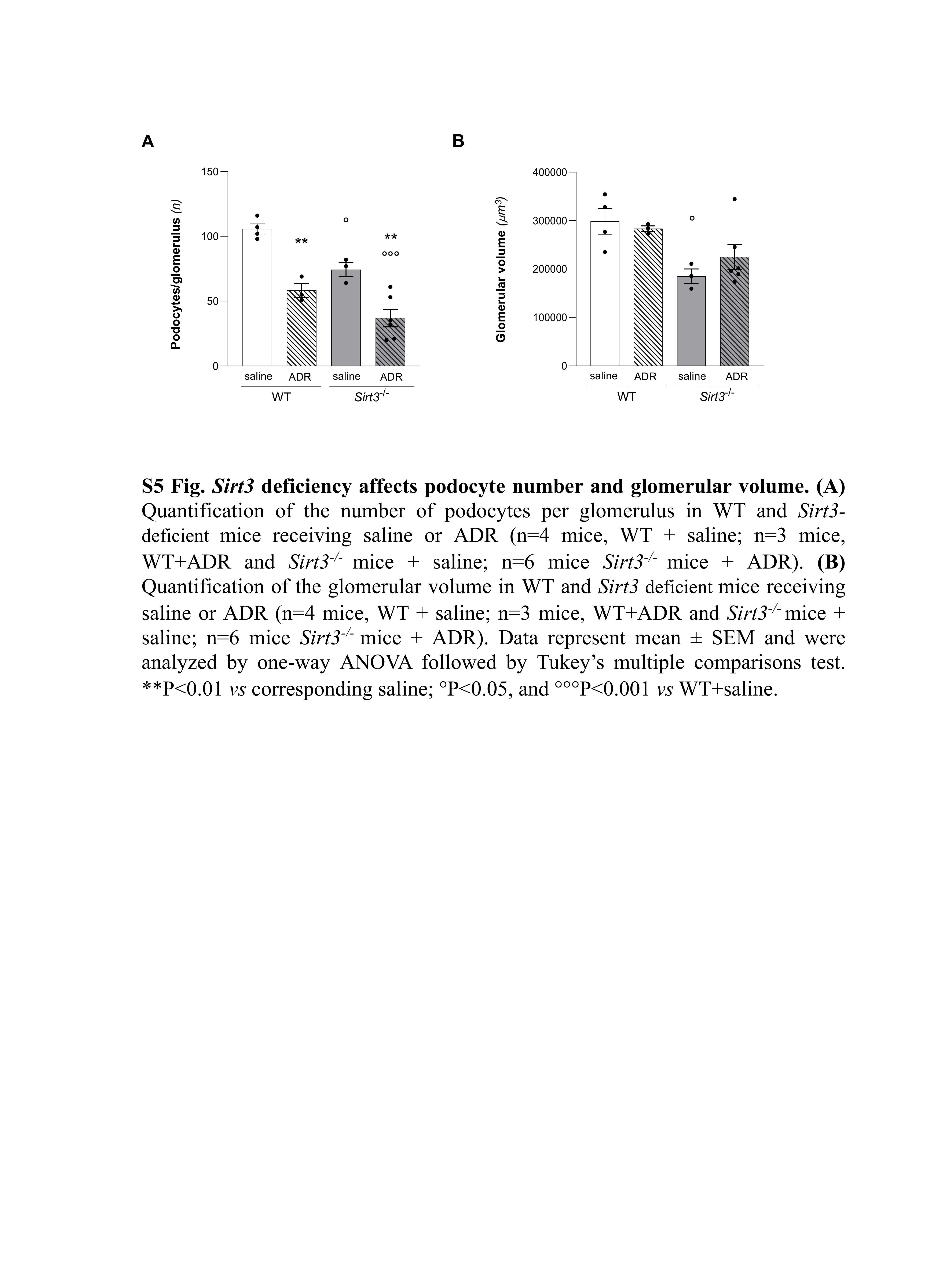

Supplement: S5 Fig — (A) Quantification of the number of podocytes per glomerulus in WT and Sirt3-deficient mice receiving saline or ADR (n = 4 mice, WT + saline; n = 3 mice, WT+ADR and Sirt3-/- mice + saline; n = 6 mice Sirt3-/- mice + ADR). (B) Quantification of the glomerular volume in WT and Sirt3 deficient mice receiving saline or ADR (n = 4 mice, WT + saline; n = 3 mice, WT+ADR and Sirt3-/- mice + saline; n = 6 mice Sirt3-/- mice + ADR). Data represent mean ± SEM and were analyzed by one-way ANOVA followed by Tukey’s multiple comparisons test. **P<0.01 vs corresponding saline; °P<0.05, and °°°P<0.001 vs WT+saline. (TIF) [file pone.0291909.s005.tif]
