# Supplementary material for: Sirt3 deficiency promotes endothelial dysfunction and aggravates renal injury
Source: PLoS One. 2023 Oct 10;18(10):e0291909. doi: 10.1371/journal.pone.0291909 (PMC10564163; doi:10.1371/journal.pone.0291909)
Supplement: S1 Raw images — All images were acquired on Odyssey FC Imaging System (LiCor, Lincoln, Nebraska, USA). In each blot, α-tubulin was used as sample loading control. Molecular weights (MW) are reported for each gel and expressed in kilo Dalton (kDa). X indicates lanes not included in the final figures. Abbreviations: WT, wild type mice; Sirt3-/-, Sirt3 knockout mice; ADR, adriamycin; VEGFA, vascular endothelial growth factor A; Angpt2, angiopoietin 2; SOD2AcK68, SOD2 acetylated at lysine 68; L-SIRT3, long SIRT3 isoform; S-SIRT3, short SIRT3 isoform (mitochondria). (PDF) [file pone.0291909.s006.pdf]

A - Uncropped blot images of VEGFA expression shown in Fig 2A.

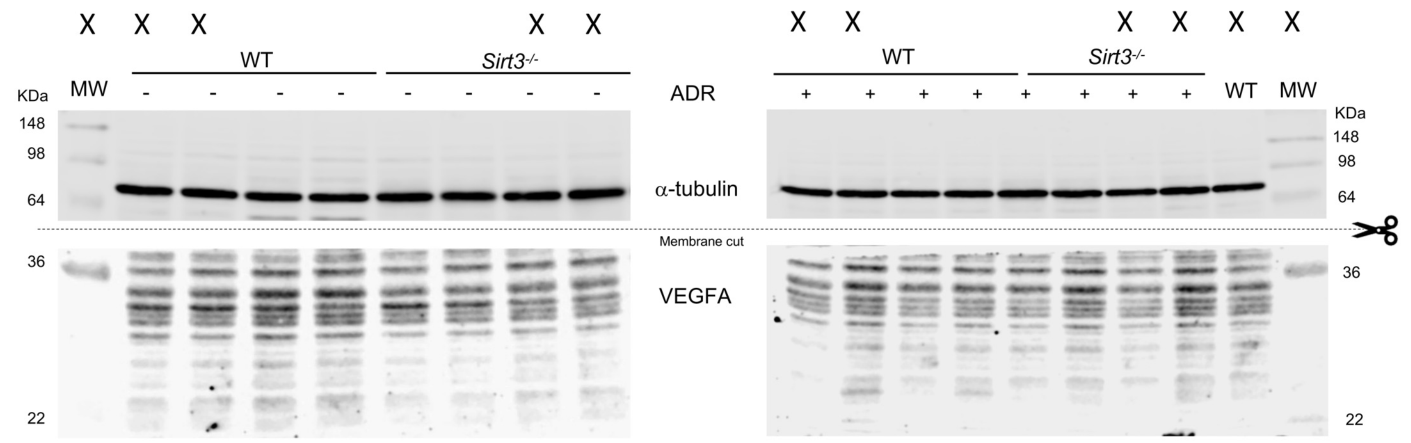

B - Uncropped blot images of Angpt2 expression shown in Fig 4B.

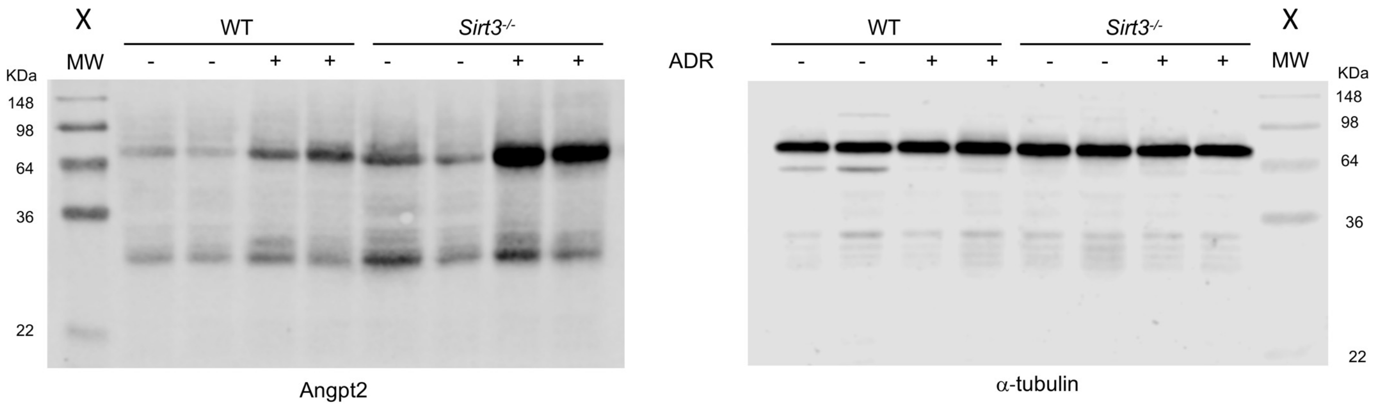

C - Uncropped blot images of SOD2^{AcK68} expression shown in Fig 4D.

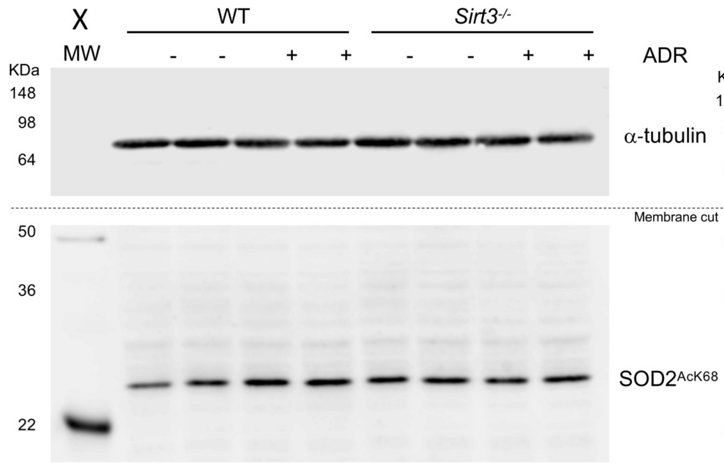

D - Uncropped blot images of SIRT3 expression shown in Fig S4.

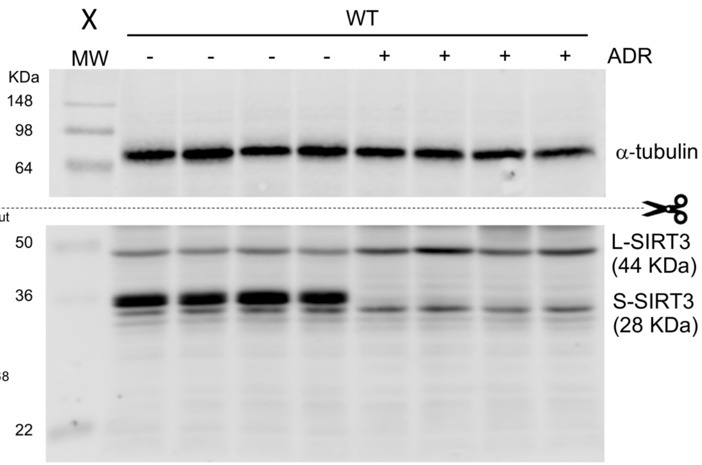

S1 raw images. Uncropped blot images included in the manuscript's main figures and supplemental figures. All images were acquired on Odyssey FC Imaging System (LiCor, Lincoln, Nebraska, USA). In each blot, α-tubulin was used as sample loading control. Molecular weights (MW) are reported for each gel and expressed in kilo Dalton (kDa). X indicates lanes not included in the final figures. Abbreviations: WT, wild type mice; *Sirt3*$^{-/-}$, *Sirt3* knockout mice; ADR, adriamycin; VEGFA, vascular endothelial growth factor A; Angpt2, angiopoietin 2; SOD2^{AcK68}, SOD2 acetylated at lysine 68; L-SIRT3, long SIRT3 isoform; S-SIRT3, short SIRT3 isoform (mitochondria).
